# Advances and challenges in skeletal muscle angiogenesis

**DOI:** 10.1152/ajpheart.00635.2015

**Published:** 2015-11-25

**Authors:** I. Mark Olfert, Oliver Baum, Ylva Hellsten, Stuart Egginton

**Affiliations:** ^1^Center for Cardiovascular and Respiratory Sciences and Division of Exercise Physiology, West Virginia University School of Medicine, Morgantown, West Virginia;; ^2^Institute of Anatomy, University of Bern, Bern, Switzerland;; ^3^Integrative Physiology Group, Department of Nutrition, Exercise and Sports, University of Copenhagen, Copenhagen, Denmark; and; ^4^School of Biomedical Sciences, University of Leeds, Leeds, United Kingdom

**Keywords:** exercise, human, rodent, training

## Abstract

The role of capillaries is to serve as the interface for delivery of oxygen and removal of metabolites to/from tissues. During the past decade there has been a proliferation of studies that have advanced our understanding of angiogenesis, demonstrating that tissue capillary supply is under strict control during health but poorly controlled in disease, resulting in either excessive capillary growth (pathological angiogenesis) or losses in capillarity (rarefaction). Given that skeletal muscle comprises nearly 40% of body mass in humans, skeletal muscle capillary density has a significant impact on metabolism, endocrine function, and locomotion and is tightly regulated at many different levels. Skeletal muscle is also high adaptable and thus one of the few organ systems that can be experimentally manipulated (e.g., by exercise) to study physiological regulation of angiogenesis. This review will focus on the methodological concerns that have arisen in determining skeletal muscle capillarity and highlight the concepts that are reshaping our understanding of the angio-adaptation process. We also summarize selected new findings (physical influences, molecular changes, and ultrastructural rearrangement of capillaries) that identify areas of future research with the greatest potential to expand our understanding of how angiogenesis is normally regulated, and that may also help to better understand conditions of uncontrolled (pathological) angiogenesis.

this article is part of a collection on **Pan American Congress of Physiological Sciences Meeting 2014**. Other articles appearing in this collection, as well as a full archive of all collections, can be found online at http://ajpheart.physiology.org/.

## Introduction

There is wide recognition (17, 38, 126) and a surfeit of epidemiologic evidence that links the benefits of exercise training with a decreased risk of cardiovascular diseases. The reasons are manifold, but one important factor is that the peripheral microvasculature adapts in structure and function to this stimulus. The role of the microcirculation, in particular of the capillaries, is to serve as the interface for delivery of oxygen and removal of metabolites to/from tissues. In this context, the assumption has always been that “more is better” and that greater capillary surface area results in increased potential for diffusion of oxygen and metabolite clearance, thereby improving endurance and aerobic capacity (65). Indeed, the literature supports a correlation between capillary density (CD) and the proportion of oxidative fibers or average mitochondrial volume density among different skeletal muscles (63), and exercise performance or maximal aerobic capacity correlate well with skeletal muscle CD in animals (65).

Given that skeletal muscle comprises nearly 40% of body mass in humans, altering muscle CD has a significant impact on metabolism, endocrine function, and locomotion. Whereas an increased skeletal muscle CD likely indicates capillary growth (angiogenesis) linked with functional improvements, decreased skeletal muscle CD (i.e., capillary rarefaction) is associated with poor health outcomes and declining prognosis in association with many chronic diseases, such as peripheral arterial disease (77, 87), diabetes (79, 80), cachexia (7), and chronic obstructive pulmonary disease (74). It is therefore important to understand the mechanisms involved in the regulation of muscle capillaries to both optimize physiological outcomes and ameliorate pathological consequences.

In contrast to vasculogenesis, which is the de novo formation of blood vessels essential in the initial development of the microcirculation in embryos, angiogenesis is the formation of capillaries from existing blood vessels. Angiogenesis plays a key role throughout all stages of life (e.g., tissue injury/wound repair, the endometrial cycle, and striated muscle adaptation to stress/exercise). During the past decade there has been a proliferation of studies that have advanced our understanding of the angiogenic processes, demonstrating that capillary growth in skeletal muscle is a process that is tightly regulated at many different levels [see reviews (29, 59, 101)]. This regulation follows the principles of a control circuit. For example, when the microcirculation is inadequate to fully meet the metabolic demands imposed on the muscle by cellular and physical influences (see below), these influences exert an upstream effect on the capillary network to proliferate (upstream input). The physical influence(s) are sensed by muscle fibers and endothelial cells (ECs) and translated into an appropriate gene expression profile (control variable), which ultimately alter the EC phenotype (downstream output) and lead to capillary growth. Angiogenesis is thought to terminate once metabolic homeostasis is reestablished (feedback). This, however, is a multifactorial and highly orchestrated process for which we are only beginning to understand the molecular underpinnings.

This review will focus on the methodology to determine CD and highlight the concepts in health and disease that are reshaping our understanding of the angioadaptation process. We will use an integrative review to assess the impact of, but also responses to, altered muscle activity and summarize selected new findings that identify areas of future research with the greatest potential to expand our understanding of the regulation of skeletal muscle angiogenesis and thus improve therapeutic efficacy.

## Methodology

As the number of studies on skeletal muscle angiogenesis continues to grow, it is increasingly important to have consistency and methodological commonality in quantitative determination of CD, to avoid confounding technical and analytical issues that can produce ambiguous results. Among the issues to consider are *1*) the direction of skeletal muscle sectioning–it is critically important to have a reference orientation, but this is sometimes neglected; *2*) appropriate structural indicators for characterization of capillarity; and *3*) regional heterogeneity of fiber type and therefore muscle capillarity within a given muscle, which can lead to unintentional bias in the capillary data reported. The latter is perhaps most widely recognized within the gastrocnemius (15) and tibialis anterior muscles (which are well known “mixed muscle” phenotypes), but all skeletal muscle exhibit varying degrees of muscle fiber type and capillary heterogeneity (5, 27, 30, 111).

**Fig. 1.**
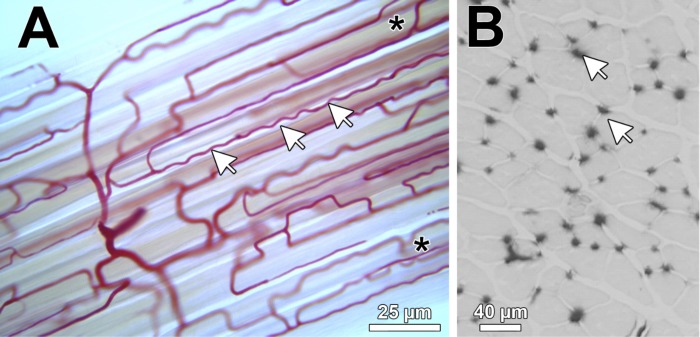
Light microscopic representation of capillaries in skeletal muscle. *A*: 60-μm-thick longitudinal section of a porcine skeletal muscle injected with colored gelatin. Note the tortuous course of the capillaries (arrows) and anastomoses (asterisks). *B*: light microscopic representation of a 10-μm-thick cryosection from a mouse extensor digitorum longus subjected to alkaline phosphatase histochemistry. The capillaries are visualized as black dots (arrows) surrounding the skeletal muscle fibers.

Skeletal muscle is an anisotropic tissue, meaning its major cellular components are not randomly arranged. The microvasculature run largely in parallel with the longitudinal axis of muscle fibers. However, the two entities may be loosely connected, with capillaries meandering over the surface of fibers (Fig. 1*A*). Hence, a potentially greater functional relevant index may be achieved by calculating capillary length density [the length of capillaries per unit volume of muscle fibers, in stereological terminology denoted as J_V_(c,f)], as this takes into account the additional surface area of each capillary offered by greater tortuosity (91). This is 7–64% higher than that estimated from CD alone in transverse tissue sections due to the tortuosity and branching anastomoses seen in skeletal muscles (90, 91). However, such quantification requires a more time-consuming and technically demanding methodology than routine estimates of CD, so unfortunately it is not commonly performed. When only muscle cross sections are evaluated, as is most often the case, appropriate values are most readily obtained with true transverse sections (i.e., perpendicular to the muscle fiber axis, as shown in Fig. 1*B*). It is important to avoid oblique sections, which lead to erroneously high-fiber area and/or counting multiple points of the same tortuous capillary undulating in and out of the horizontal plan of the muscle section. However, it should be noted that with an appropriate sampling design, it is possible to get estimates of CD from longitudinal sections, but this too is not often performed.

The number of muscle fiber and capillary profiles within a given area can be used to calculate the numerical capillary-to-fiber ratio (C:F) [in stereological notation, N_N_(c,f)]. This should not be confused with the number of capillaries surrounding individual fibers [i.e., N(c,f) or N_CAF_] or the derived index of capillary contacts, which are both quite insensitive to changes during tissue remodeling and hence have limited utility as a quantitative index of angiogenesis (30). In addition, the number of capillary profiles per cross-sectional area of muscle fibers is used to determine the CD [i.e., capillaries mm^−2^]. This measure is also frequently referred to as microvessel density, which can add ambiguity to the literature when studies include terminal arterioles and collecting venules within this definition. In stereological terms CD is denoted as N_A_(c,f) in striated muscle and provides a useful index of the functional capillary supply. However, it is important to recognize that CD depends on the mean cross-sectional area of muscle fibers. As mean cross-sectional area of muscle fibers is often modulated in response to changes in physical demand or the metabolic environment, CD should not be relied on as the sole metric used to quantify the extent of angiogenesis. Rather, inclusion of the C:F is often recommended since it is less sensitive to the issue of scaling (32).

**Fig. 2.**
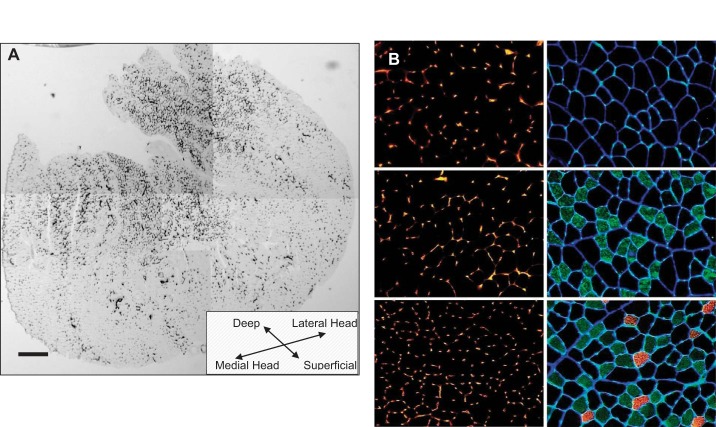
Capillary and fiber-type heterogeneity within skeletal muscle. Depicted here is capillary heterogeneity within the gastrocnemius (*A*) and extensor digitorum longus (*B*) skeletal muscle. *A*: montage of 5 overlapping images obtained using light microscopy (5×) to depict the entire transverse (or horizontal) view of a single mouse gastrocnemius muscle stained with alkaline phosphatase (capillaries appear as dark spots within the muscle section). Scale bar = 400 μm. *B*: images obtained using light microscopy (20×) from transverse section of mouse extensor digitorum longus muscle. *B*, *left*: images stained with rhodamine-labeled lectin (capillaries appear as bright spots within the muscle section). *B*, *right*: adjacent images stained for fiber types (and which are obtained from same region corresponding to images at *left*). Immunolabeling reveals fibers appearing black (dark) are type IIB, green-appearing fibers are type IIA, and orange-colored fibers are type I. Blue stain is laminin used to delineate fiber boundaries. *A* & *B*: significant heterogeneity in muscle capillarity depending on region of sampling within a muscle and that (as seen in *B*) this heterogeneity corresponds to muscle fiber-type heterogeneity found in muscles with mixed fiber-type composition. Scale bar = 200 μm. (Kissane and Egginton, unpublished.)

Finally, skeletal muscle displays a varying degree of fiber-type heterogeneity that can have a direct and significant influence on the quantitative assessment of muscle capillarity indexes. For example, the gastrocnemius and tibialis anterior muscles are frequently studied because of their active role in locomotion and exercise. But these and other skeletal muscle represent a “mixed” muscle type containing a heterogeneous distribution of myofiber phenotypes (27, 116) with concomitant regional heterogeneity in capillary supply (Fig. 2). Thus rigorous procedural attention is required when selecting histological images for counting to avoid sampling bias within any muscle sample. Indeed, unbiased counting rules defining randomness of sampling and counting procedures are essential and obviate the need to analyze the entire muscle when the rules of stereology are strictly followed (31, 34).

## Physical Influences

**Fig. 3.**
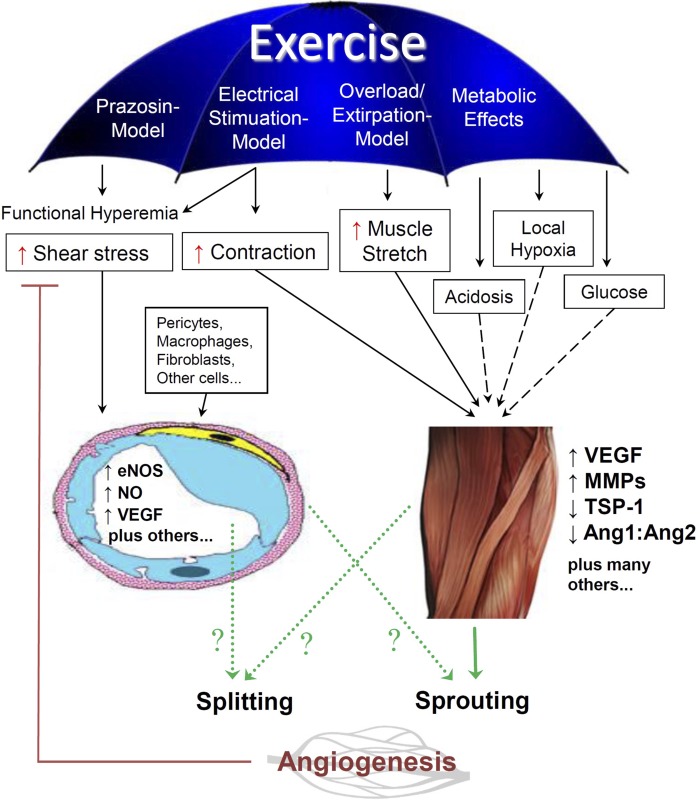
Overview of the physical and metabolic stimuli contributing to angiogenesis in skeletal muscle in response to endurance exercise. Exercise leads to an increase in blood flow through capillaries (functional hyperemia), as well as metabolic and mechanical (physical) alterations of the skeletal muscle fibers that may modulate the gene expression patterns of angiogenic factors, and subsequently, change the capillary ultrastructure (e.g., pericytes, protrusions, etc.) that determines angiotype (e.g., splitting and sprouting angiogenesis) in skeletal muscle. Solid lines (–––) represent the physical stimuli that are believed to influence exercise-induced skeletal muscle angiogenesis. Dashed lines (− − −) represent metabolic stimuli that have also been reported to have either primary or secondary influences on exercise-induced skeletal muscle angiogenesis. Dotted green lines (···) represent the potential sources releasing molecular factors responsible for angiogenesis. eNOS, endothelial nitric oxide (NO) synthase; MMP, matrix metalloproteinase; TSP-1, thrombospondin-1.

Physiological regulation of CD (i.e., during adaptive remodeling) involves a significant influence of the physical environment (Fig. 3). For example, mechanical factors such as increased shear stress exerted on the capillary lumen by functional hyperemia, or elevated tissue strain as a consequence of repetitive contraction/relaxation cycles may stimulate capillary growth in both cardiac and skeletal muscle (65).

The in vivo control of angiogenesis involves a complex array of stimuli and an ever-growing multifactorial list of candidates associated with muscle exercise activity [see reviews (29, 59, 101)], which invites a reductionist approach to better define potential contributors. Functional hyperemia increases microvascular shear stress (because of low compliance of capillaries) and chronically elevated blood flow is capable of inducing angiogenesis in the absence of any change in muscle phenotype, which demonstrates a potential disconnection between the cellular feedback from host tissue that has usually been deemed essential for angiogenesis. For example, when muscles were exposed to targeted hindlimb dilatation, raising average capillary wall shear stress by nearly fourfold, there was a 30% increase in C:F (24).

At rest, blood flow to skeletal muscle is low, but during contractile activity, it can be enhanced up to 100-fold to accommodate the large increase in oxygen demand (3). Blood flow to the muscle is determined by perfusion pressure and vascular conductance. The latter is well balanced and regulated by sympathetic vasoconstriction, sympatholytic compounds, and vasodilators, which are locally produced in the muscle (primarily by EC, red blood cells, and skeletal muscle fibers). Regulation of vascular conductance involves flow-mediated, conducted, and red blood cell-mediated dilatation, as well as interaction between several vasodilators, including nitric oxide (NO), prostacyclin, adenosine, and ATP (114). NO and prostaglandins have been found to be central in blood flow regulation, as the vasodilatory effect of many other compounds (e.g., acetylcholine, adenosine, ATP, and bradykinin) as well as mechanical signals (shear stress) are mediated through the formation of these compounds (55). The vascular system is therefore particularly sensitive to alterations in NO and prostaglandin bioavailability. This may be particularly important in the context of capillary adaptation, since negative angiogenic regulators, such as thrombospondin-1 (TSP-1), which is discussed later, are also known to inhibit NO activity (71, 109).

The large increase in blood flow to muscle during exercise will induce a significant increase in shear stress in the capillary bed and thereby promote angiogenesis. Similarly, several vasodilators (including NO, prostaglandins, and adenosine) are also implicated in the regulation of muscle angiogenesis (1, 8, 106), e.g., NO simultaneously induces vasodilatation and enhances vascular endothelial growth factor (VEGF) expression (11). Not surprisingly, there is a close association between the magnitude of blood flow in tissues and the level of capillarization (127). In addition to the usual increase in VEGF, high shear stress also leads to upregulation of endothelial NO synthase (eNOS) mRNA and protein expression, possibly leading to feedback promotion of VEGF expression (8). However, the level of shear stress induced by pharmacological vasodilatation is likely in excess of that associated with functional hyperemia induced by electrical stimulation in rodents. But, it must be noted that direct measurements of capillary shear stress during exercise in humans are lacking.

Muscle stretch, either during normal duty cycle or overload (induced with synergistic muscle extirpation), is also a potent angiogenic stimulus accompanying muscle hypertrophy (25, 121). In resistance training mitochondrial volume density decreases in proportion to muscle hypertrophy, whereas with stretch, capillary supply increases in response to an anabolic (aerobic) environment caused by imposition of a sustained strain of 20%, where C:F increased by some 45% (131). In this case higher VEGF levels were accompanied by elevated matrix metalloproteinase (MMP) activity associated with extracellular matrix remodeling (49). Interestingly, MMP inhibition prevented EC migration but not proliferation during muscle stimulation, demonstrating differential control over individual elements of the angiogenic cascade (32).

Indirect electrical stimulation of skeletal muscle is one of the most potent angiogenic stimuli known, and in rodent hindlimb extensors, it can increase C:F by 50% (36). In this experimental model, the multifactorial consequences of such activity–hemodynamic forces (shear stress, transmural pressure) and dynamic duty cycles (muscle stretch and compression)–initiate remodeling of the vasculature by activating endothelial mechanotransduction mechanisms. These are likely mediated by integrins and associated GTPases. Subsequent signal transduction acts through phosphorylation of kinases (e.g., FAK, c-Src, Akt kinase, phosphatidylinositol 3-kinase, MLCK, and MAPK) to elicit a response appropriate to the stimulus and tissue (29). But it should also be noted that electrical stimulation and its indirect effect of increasing shear stress can result in damage to the luminal glycocalyx that may also potentiate the endothelial response (32). Nevertheless, it is thought that the angiogenic stimuli elicited via electrical stimulation is likely the most similar to that experienced during endurance exercise.

There is great potential to exploit these findings in developing specific angiotherapies, in particular the differential response of muscle and microcirculation. However, there is a growing awareness of the need to better understand the time course of adaptation and/or the intensity of the stimulus. For example, whereas forced treadmill running typically requires at least 6 wk to elicit a significant increase in skeletal muscle angiogenesis, voluntary wheel running in mice has been shown to increase skeletal muscle CD starting after only 5–7 days (99). Likewise, functional hyperemia and overload stimuli achieve similar responses in 2 wk, whereas electrical stimulation can do so in only 1 wk (32). Based on data from whole body exercise, it is becoming evident that the volume of exercise (i.e., time spent exercising) also has an influence capillary adaptation that is currently underappreciated. For example, rodents given access to running wheels voluntarily run between 5–10 km/day and rarely exceed 25% of their maximal effort, but yet achieve capillary expansion rapidly (i.e., 5–7 days). In contrast, forced treadmill running regimes typically attain <2 km/day but often impose exercise above 50% of maximum effort and usually take 6 wk or more for changes in muscle capillarity to occur. These data suggest that the combination of high-volume, low-intensity exercise (i.e., voluntary wheel running) elicits a quicker angioadaptive response than that occurring with low-volume, high-intensity exercise programs. Similar time course experiments are not available in humans, but training studies involving different exercise intensities indicate that the angiogenic response to a period of training at moderate intensity is similar to that of high-intensity training in previously untrained individuals (21, 60, 72). However, there are indications that very high-intensity training can actually lead to a negative effect on VEGF levels (43, 61). In addition, the recognition that mechanical factors may play as potent a role in microvascular remodeling as chemical or metabolic factors (such as local tissue hypoxia, acidosis, glucose, etc.) has led to the concept of “passive” training, which shows an elevation of angiogenic factor expression in muscle without active work being performed (56). Thus it is tempting to speculate that low-intensity, high-volume exercise regimes may produce a stronger angiogenic stimulus and perhaps use intussusception/longitudinal splitting (i.e., a mode of angiogenesis with less energetic requirements compared with sprouting, discussed further in following section) and therefore result in more rapid capillary adaptation compared with capillary sprouting (that is expected from high-intensity, low-volume exercise frequently used with forced treadmill running in rodents). At present, however, the potential differential stimuli and associated molecular responses between exercise modalities are still only poorly understood and will require more deliberate investigation to unravel. Indeed, future studies aimed at directly assessing these responses are needed, as they may also help to devise and improve modes of exercise to maximize the benefits from rehabilitation therapies in the clinic.

## Capillary Ultrastructure

### Endothelial cells.

**Fig. 4.**
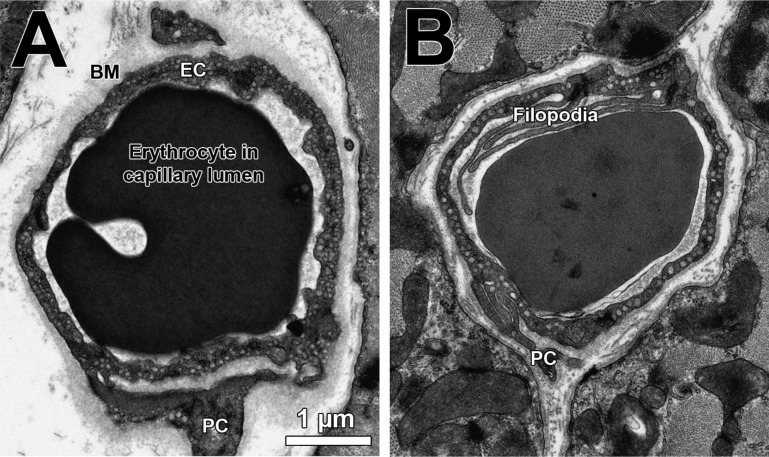
Characteristic ultrastructure of a capillary from human (*A*) and mouse (*B*) skeletal muscles depicted by transmission electron microscopy. In general, capillaries from humans are larger, exhibit a thicker basement membrane (BM), and form less filopoda than those from mice. EC, endothelial cell; PC, pericytes. Same scale applies to both images.

Microvessel in skeletal muscles manifest a characteristic ultrastructure, which is best seen by transmission electron microscopy. Shown in Fig. 4 is a transmission electron microscopy image where the outer (abluminal) surface of capillary ECs is covered by a dense basement membrane frequently containing profiles of one or more perivascular pericytes. It is interesting to note, this image appears to show that both cell types communicate extensively during angiogenesis (9, 66). In response to elevated luminal shear stress (prazosin-treated rats), a higher proportion of intraluminal irregularities, projections, and septa combined with extensive cytoplasmic vacuolization of EC were observed in skeletal muscle capillaries, which are interpreted as signs of a longitudinal splitting form of angiogenesis with little evidence for EC proliferation (130). When the canonical sprouting angiogenesis was induced in rats by increased stretch of skeletal muscles due to overload (131) or during chronic electrical stimulation (51), the proportion of abluminal sprouts was higher in capillaries during active angiogenesis, whereas the labeling index (bromodeoxyuridine incorporation) was higher than in the control animals, suggesting that this angiotype is associated with proliferation of ECs (131). In addition, endurance exercise is accompanied by thinning of the pericapillary basement membrane (125), which is presumably induced by the higher expression/activity of MMPs and their proteolytic actions (110). Indeed, in studies with rodents, hallmarks for changes in the ultrastructural phenotype of capillaries in response to angiogenic stimuli have been identified (36).

While the sprouting form of angiogenesis is characterized by hallmarks of EC proliferation and migration (110), nonsprouting angiogenesis can be divided into *1*) longitudinal splitting, involving cytoplasmic invagination and bridging over the lumen, and *2*) intussusception that occurs with infiltration of stromal cells (36). It is thought that as nonsprouting angiogenesis involves a much lower extent of EC proliferation, this may be an energetically efficient and potentially more rapid way of extending the microcirculation.

Surprisingly, it has not yet been determined whether sprouting or splitting/intussusception is used to realize physiological angiogenesis in skeletal muscles in response to exercise or whether this could explain the temporal difference in capillary adaptation between voluntary wheel running versus forced treadmill exercise. It is interesting to note that EC production of VEGF appears not to be essential to elicit angiogenesis, since selective deletion of VEGF in ECs does not appear to alter CD in a variety of organs (83). However, it should be emphasized that tight regulation of angiogenic activity still requires involvement of EC, as well as influence of perivascular cell types within the interstitium. Indeed, this cell-cell contact suggests other signaling routes may be operative (32), perhaps in the context of basement membrane remodeling, potentially balancing activity of angiogenic inhibitors and stimulators.

### Pericytes.

Because of their close spatial relationship to ECs, it was speculated that pericytes contribute to the regulation of angiogenesis (41, 57), involving a combination of tight junction electrical communication and paracrine influence, e.g., VEGF (62). Indeed, there was a significant reduction in pericyte coverage of ECs during early phases of angiogenesis in skeletal muscles of rats subjected to chronic electrical stimulation, which suggested an antiangiogenic impact of pericytes in this experimental model (33). In contrast, long-term peripheral vasodilation (prazosin model) or prolonged stretch (extirpation model) was accompanied by no change or an increase in pericytes coverage, respectively (35). Thus the role of pericytes in controlling physiological angiogenesis seems context dependent, reflecting the nature of the initial stimulus (35), although the contribution of pericytes subtypes to different angiotypes has yet to be characterized (12).

### Fibroblasts, mast cells, macrophages, and circulating cells.

Mesenchymal fibroblasts are cells within the interstitium that synthesize the ECM and connective tissue of the stroma. They are perhaps best known as the origin of pathological conditions such as fibrosis but also aid inflammation and tumor growth. Their physiological role is to support cell-cell interaction and cell migration and may mediate angiogenesis chemically (via expression of angiogenic factors) and mechanically (via disruption of connective tissue and ECM deposition) (67). In sprouting angiogenesis, the number of fibroblasts increases in close proximity to sites of neovascularization following increased muscle activity and are responsible for much of the mitotic activity evident with light microscopy (36), although whether activated EC attract motile fibroblasts or activated fibroblasts stimulate an EC response remains unclear (32).

Mast cells of the hematopoietic lineage are terminally differentiated in peripheral tissue, where activation causes release of growth factors and inflammatory cytokines that may stimulate angiogenesis in a pathological environment such as found in tumors. In muscle they are found within the interstitial, often close to capillaries, where they are thought to be responsive to both mechanical deformation and/or metabolite release. Although mast cells may be important in cardiac muscle remodeling, their activation appears not to be an important regulator of angiogenesis as a result of increased skeletal muscle activity (28).

Similarly, macrophages are ubiquitous constituents of tissue but are also thought to mainly play a role in inflammation-mediated angiogenesis, as a result of secreted factors under pathological conditions. However, the resident population may be renewed by turnover or by infiltration of other macrophages. Interestingly, an increase in these VEGF-positive cells within the interstitium appears to be an early sign of angiogenesis in response to increased muscle activity (18), perhaps an indication that part of the exercise-induced remodeling has an inflammatory basis (107).

There is increasing interest in the potential role of circulating cells within the local vasculature for regulation of angiogenesis. Perhaps the best known are the hematopoietic stem cells that support vigorous angiogenesis within solid tumors and may supplement endothelial turnover during tissue repair. Other cell types will require further investigation. However, increased leukocyte concentration around venules (the supposed site of new capillary growth) within skeletal muscle suggest they may have some regulatory influence, but in physiological angiogenesis this appears to be unlikely (105). Intriguingly, however, circulating platelets appear to have a differential effect on physiological angiogenesis within skeletal muscle, likely influencing endothelial migration by cyclooxygenase signaling (105). Platelets are also a major site for the production of TSP-1, which exerts a negative influence on EC production and migration (19), but thus far there is little evidence that circulating plasma/serum TSP-1 levels have any direct influence on skeletal muscle angiogenesis.

## Molecular Changes

The orchestration of molecular information at various levels is required to generate the appropriate extent of angiogenesis in skeletal muscles. Although details are still to be finalized, it is evident that this involves sensitive feedback control and disruption of such signaling is likely a component of hyperproliferative or ischemic vascular diseases. There is an ever-growing list of pro- and antiangiogenic factors that must remain in balance to effect microvascular homeostasis [see reviews (53, 101)]. We will focus on the description of those that have emerged as the major players and some new factors (Table 1) that may provide significant impact on our understanding of physiologically controlled angiogenesis. This does not diminish the potential importance of the factors not discussed here but points out that our current understanding for many of the positive and negative angiogenic regulators remains circumstantial and in some cases context dependent.

**Table 1.** Emerging regulators and their actions and responses

Emerging Regulators	Action/Response	References
FoxO1	Modulate TSP-1 effects	(94, 112, 118)
Nucleolin	Interacts wiht VEGF and MMP9.	(37, 64)
	EC surface receptor for endostatin.	(97, 98, 117)
Ephrin B2	Increased by stretch and hypoxic stress.	(75, 128)
Polymerae δ-interacting protein 2 (Poldip2)	Necessary for vascular integerity, EC proliferation, inhibition of MMPs, and cell adhesion.	(120)
PGC-1α and Secreted phosphoprotein 1	Transcriptional coactivator responsible in the function of mitochondrial biology, electron transport chain, δ oxidation, and influence on angioregulatory mitogens (e.g., VEGF).	(4, 96, 112)
Vasoinhibin-1	Anti-angiogenic mitogen	(76, 78, 101)
	Promotes vessel stablization and maturation.	
Endosialin (CD248)	Required for PDGFRβ-dependent capillary sprouting angiogenesis.	(95)

FoxO1, Forkhead Box O 1; TSP-1, thrombospondin-1; MMP, matrix metalloproteinase; EC, endothelial cell; PDGFR, PDGF receptor.

### Vascular endothelial growth factor.

VEGF exists in several homodimeric isoforms: VEGF_121_, VEGF_145_, VEGF_165_, VEGF_189_, and VEGF_206_ (85). Although several of these isoforms likely play a role in angiogenesis, VEGF_165_ has been found to be the most central proangiogenic factor in skeletal muscle. Some of the early evidence for the importance of VEGF in angiogenesis was that the training-induced increase in capillary growth was partially reduced with VEGF receptor blockade (86). Moreover, treatment of rodents with a VEGF sequestor revealed that VEGF is required for both shear stress- and overload-induced angiogenesis (124). Studies on genetically modified mice lacking muscle-specific VEGF have demonstrated that basal capillarization is markedly reduced compared with wild-type mice (103) and that the training-induced increases in capillary growth is abolished (26, 104), providing solid evidence for the importance of VEGF in muscle. Additional studies using myocyte-specific, VEGF-deficient mice revealed that skeletal muscle angiogenesis does not occur in response to prazosin-mediated shear stress (122) or extirpation-mediated muscle stretch (i.e., muscle overload) (45), which seems to clearly establish that muscle fiber-derived VEGF is essential to initiate the angiogenic process within skeletal muscle.

An acute exercise bout leads to a transient, severalfold increase in expression of VEGF mRNA (16, 48, 108) with levels returning to baseline after 4–6 h of recovery (46, 73). Levels of VEGF protein also increase after an acute exercise bout in rodents (99) and are elevated during the first few weeks of aerobic exercise training in young healthy individuals (47) and animals (99), whereas in humans VEGF protein levels appear to return to baseline after 4 wk of training (60). This pattern of VEGF protein changes appears to be different in individuals with lifestyle-related diseases (52) and ageing (22, 44), where muscle VEGF levels are lower in elderly than in young individuals at baseline but are enhanced with exercise training.

For muscle fiber-derived VEGF to promote angiogenesis, secretion to the extracellular space is required, and mechanical stimulus induced by passive movement of the muscle (56) or active muscle contraction (39, 58) leads to increased muscle interstitium VEGF levels. While increases in extracellular VEGF can originate from different cell types including ECs, fibroblast, and pericytes, evidence (noted above) points to myocytes being the most important source (62, 73). Muscle fibers contain high levels of VEGF stored in vesicles (62), and synthesis of VEGF does not appear to be required for secretion but likely occurs after contraction to replenish muscle stores (62). The exercise-induced increase in muscle interstitial VEGF does not appear to be altered by exercise training in healthy individuals (39, 60) but may be improved by training in individuals with lifestyle-related disease (52). At present, the mechanisms underlying VEGF secretion from skeletal muscle, and how this is altered by disease and exercise, has yet to be fully determined. While there is robust evidence that VEGF is required for the angiogenesis process, it must be emphasized that VEGF cannot act alone, and other factors are also essential and need to be co-regulated to elicit angiogenesis.

### Nitric oxide.

The gaseous, radical NO (NO·= NO) is generated by the catalytic activity of three intracellular NO synthases (NOSs). While the existence of the inducible NOS (iNOS) in skeletal muscle is not confirmed, eNOS is found in ECs of all vascular segments including capillaries (115), whereas neuronal NOS (nNOS) is present at high concentrations in the sarcolemma of skeletal muscle fibers in close proximity to the microcirculation (119).

A modulatory impact of NO on angiogenesis has been shown in rodents. Administration of NOS inhibitors, such as *N*^G^-nitro-l-arginine (l-NNA) and *N*^ω^-nitro-l-arginine methyl ester (l-NAME), blunts angiogenesis in skeletal muscle in response to chronic electrical stimulation (35) and whole body exercise (40). Furthermore, the increased expression of VEGFR-2 and VEGF proteins in skeletal muscles was abrogated in rats electrically stimulated for 2 and 4 days but not for 7 days (93). These data implied that chronic electrical stimulation leads to induction of two peaks of VEGF and VEGFR-2 expression: an early first NO-dependent increase required for the onset of angiogenesis and a later, NO-independent rise that may aid maintenance of the expanding capillary bed (93).

However, chemical NOS inhibitors used do not typically discriminate between NOS isoforms, and understanding the differential impact of each one on angiogenic events requires studies with NOS-deficient knockout (KO) mice. The VEGF increase in ECs (23) and angiogenesis (8, 123) were blunted in skeletal muscles of eNOS-KO mice after prazosin administration, suggesting that eNOS-derived NO produced represents an obligate upstream signal for shear stress-induced angiogenesis (8). This angiotype depends on functional hyperemia and the vasodilatory potential of arteries, which is significantly lower in eNOS-KO than WT mice (81, 129). In contrast, overload-induced sprouting was not prevented in eNOS-KO mice, and there was no effect on either splitting or sprouting angiogenesis in nNOS-KO mice (10, 123). Hence, the influence of NO on expression of VEGF might depend on the site of NO at which it is generated and the context of the physical stimuli (29).

In humans, a direct role of NO for skeletal muscle angiogenesis has not been determined; however, eNOS is upregulated in muscle in response to acute exercise, exercise training, and passive movement leading to increased shear stress (21, 54, 56). A similar role for eNOS and NO in angiogenesis in humans, as seen in rodents, therefore seems likely.

### Thrombospondin-1.

The first reports that acute exercise increased, but that exercise training decreased, skeletal muscle TSP-1 expression (102) and that increasing shear stress decreased EC expression of TSP-1 (14) provided an incentive to understand the role of negative regulators of angiogenesis in this context (53, 101). Indeed, whereas a single exercise bout increased skeletal muscle TSP-1 and VEGF mRNA expression up to fourfold, repeated daily bouts of exercise reduces the TSP-1 (but not VEGF) response to exercise (102). More recently, the transcription factor Forkhead Box O 1 (FoxO1) has been shown to transcriptionally regulate EC production of TSP-1 (112). Whereas increasing FoxO1 can restrain angiogenesis in ischemic skeletal muscle (94, 112), gene deletion of FoxO1/FoxO3a abolished the increase in TSP-1 expression following acute exercise and induced a more rapid exercise-induced expansion in muscle capillarity (118). Studies examining skeletal muscle expression of TSP-1 in response to varying duration and intensity of exercise training (60, 68, 69, 82, 88, 89) indicate that it likely plays a key regulatory role in controlling the onset of exercise-induced angiogenesis. Thus, while VEGF-A is perhaps the most central angiogenic mitogen whose secretion is an absolute requirement for the initiation of skeletal muscle angiogenesis, it appears that TSP-1 may be essential for the necessary physiological constraint on capillary expansion (100).

Responses following physical deconditioning (i.e., detraining) in rodents demonstrate that capillary regression seems to occur in temporal correlation to muscle TSP-1 protein expression, even in the face of elevated muscle VEGF expression (97). Hence, while VEGF is necessary to initiate angiogenesis, it appears that TSP-1 (and likely other antiangiogenic factors) may exert principal control on the growth process, determining when and possibly where angiogenesis takes place. Indeed, there is also ample evidence from gain and loss of function studies involving TSP-1 that support the importance of TSP-1 in regulating muscle angiogenesis under both physiological (6, 89) and pathological (70, 80, 92) conditions.

### Other pro- and antiangiogenic factors.

While much attention has focused on VEGF-A, and increasingly TSP-1, numerous other factors are known to exhibit either proangiogenic or anti-angiogenic properties in other tissue. These include growth factor/cognate receptor pairings such as the angiopoietins/TIE receptors, ephrins/Ephs, FGFs/FGF receptors, PDGFs/PDGF receptors, and delta-like ligand 4/notch, in addition to endothelial function modulators such as nNOS and eNOS. There is a growing realization that these factors need to be presented in spatially and temporally balanced sequences for the formation and maintenance of functional vessels in skeletal muscle [reviewed in Olfert and Birot (101)]. Furthermore, various proteins without previously established function in vascular biology, such as secretion of phosphoprotein 1 (113), polymerase δ-interacting protein 2 (2), nucleolin (97, 98), as well as peroxisome proliferator-activated receptor-γ coactivator 1α (PGC-1α), all appear to have influence on capillarity in skeletal muscles.

Notably, PGC-1α is a key transcriptional coactivator that regulates expression of genes involved in mitochondrial biogenesis and energy metabolism (50, 99). Recent evidence from muscle-specific PGC-1α KO mice suggest that this factor is an important mediator of exercise-induced angiogenesis (4). The molecular mechanisms behind this effect include exercise-dependent β-adrenergic signaling that induces transcription of an additional isoform (PGC-1α2) from an alternative promoter, which in turn increased expression of angiogenic factors such as VEGF-A and angiopoietin 2 (4, 20, 84). The use of this alternative PGC-1α promotor also seems important for the response of skeletal muscle to exercise in humans (96). Angiogenic activity of PGC-1α might involve its downstream ligand peroxisome proliferator-activated receptor-β/δ (13). Translation of PGC-1α2 mRNA leads to two PGC-1α protein variants with slightly different NH_2_-terminus (PGC-1α2 and PGC-1α3) (20). A recent study suggests that PGC-1α2 is the most responsive to a single exercise bout in human skeletal muscle (42). Despite remaining questions, it is clear that PGC-1α and its isoforms likely play an important role in regulating skeletal muscle angiogenesis.

Notwithstanding that the expanding list of factors that are now known to have an impact on this physiological process, their roles often remain only partially understood and need to be characterized in more detail. A vital and important arena for future research is the specific roles and particularly the interplay of these gene products during angiogenesis in skeletal muscle.

## Summary and Relevance

Beyond growth and development, physiologically controlled angiogenesis is largely limited to *1*) the female reproductive cycle, i.e., menses, involving smooth muscle, and *2*) skeletal muscle adaptation to exercise stimuli. Therefore, studies aimed at understanding angioadaptation and regulation of angiogenesis in muscle provide a great opportunity to investigate and understand the complexity and highly structured choreography essential for controlled regulation of angiogenesis. Given the number of diseases that involve aberrant regulation of angiogenesis, resulting in either uncontrolled vessel growth or capillary rarefaction, it is imperative that a fundamental understanding of the mechanisms that work to effectively control angiogenesis is attained, before we can truly fully understand pathological dysregulation. Indeed, lessons learnt from the past decade reinforce the need for a more comprehensive understanding of the temporal control and degree of interaction between stimulators and inhibitors of angiogenesis that goes beyond single gene and protein responses. It is here that use of multiplexing technology, including but not limited to -omic levels approaches, will allow a more global assessment angiogenic-related factors which provide the greatest opportunities to fully understand the complex interplay between stimulators and inhibitors of angiogenesis. From this foundation we will be better equipped to develop tools and/or therapies that effectively combat uncontrolled vessel growth (such as that seen in cancer/tumor development) or capillary loss often associated with many chronic diseases (such as diabetes, hypertension, Alzheimer's, osteoporosis, etc.).

## GRANTS

I. M. Olfert received financial support, in part, by a grant to the West Virginia Clinical and Translational Science Institute (National Institute of General Medical Sciences Grant U54GM104942), American Heart Association Innovation Research Grant 13IRG14330015, West Virginia University Research Foundation PSCoR grant, and from West Virginia University School of Medicine. O. Baum recieved financial support by Swiss National Science Foundation Grant 320030-144167. Y. Hellsten recieved financial support from The Danish Council for Independent Research-Medical Sciences, The Lundbeck Foundation, Novo Nordisk Foundation, and the Danish Ministry of Culture. S. Egginton recieved financial support from the British Heart Foundation Grant PG/14/15/30691.

## DISCLOSURES

No conflicts of interest, financial or otherwise, are declared by the author(s).

## AUTHOR CONTRIBUTIONS

I.M.O., O.B., Y.H., and S.E. worked together on the conception, design, interpretation and critical analysis of the data and literature reviewed; I.M.O. and O.B. prepared figures; and all authors participated in drafting the manuscript and approving the final version of manuscript.
